# Chondroitin sulfate synthase 1 enhances proliferation of glioblastoma by modulating PDGFRA stability

**DOI:** 10.1038/s41389-020-0197-0

**Published:** 2020-02-04

**Authors:** Wen-Chieh Liao, Chih-Kai Liao, To-Jung Tseng, Ying-Jui Ho, Ying-Ru Chen, Kuan-Hung Lin, Te-Jen Lai, Chyn-Tair Lan, Kuo-Chen Wei, Chiung-Hui Liu

**Affiliations:** 1 Faculty of Medicine, Department of Anatomy, Chung Shan Medical University, Taichung, Taiwan; 2 Department of Medical Education, Chung Shan Medical University Hospital, Taichung, Taiwan; 3 Department of Psychology, Chung Shan Medical University, Taichung, Taiwan; 4 Institute of Medicine, Chung Shan Medical University, Taichung, Taiwan; 5 Department of Psychiatry, Chung Shan Medical University Hospital, Taichung, Taiwan; 6 School of Medicine, Chang Gung University, Taoyuan, Taiwan; 7 Department of Neurosurgery, Chang Gung Memorial Hospital, Linkou Medical Center, Taoyuan, Taiwan

**Keywords:** CNS cancer, Growth factor signalling, CNS cancer, Growth factor signalling

## Abstract

Chondroitin sulfate synthases, a family of enzyme involved in chondroitin sulfate (CS) polymerization, are dysregulated in various human malignancies, but their roles in glioma remain unclear. We performed database analysis and immunohistochemistry on human glioma tissue, to demonstrate that the expression of CHSY1 was frequently upregulated in glioma, and that it was associated with adverse clinicopathologic features, including high tumor grade and poor survival. Using a chondroitin sulfate-specific antibody, we showed that the expression of CHSY1 was significantly associated with CS formation in glioma tissue and cells. In addition, overexpression of CHSY1 in glioma cells enhanced cell viability and orthotopic tumor growth, whereas CHSY1 silencing suppressed malignant growth. Mechanistic investigations revealed that CHSY1 selectively regulates PDGFRA activation and PDGF-induced signaling in glioma cells by stabilizing PDGFRA protein levels. Inhibiting PDGFR activity with crenolanib decreased CHSY1-induced malignant characteristics of GL261 cells and prolonged survival in an orthotopic mouse model of glioma, which underlines the critical role of PDGFRA in mediating the effects of CHSY1. Taken together, these results provide information on CHSY1 expression and its role in glioma progression, and highlight novel insights into the significance of CHSY1 in PDGFRA signaling. Thus, our findings point to new molecular targets for glioma treatment.

## Introduction

Aberrant composition of glycans in tumor microenvironment and abnormal expression of extracellular matrix (ECM) protein are hallmarks of all types of malignant tumors^1,2^. Chondroitin sulfate (CS), heparan sulfate (HS), keratan sulfate, and hyaluronan belong to the glycosaminoglycan (GAG) family, and compose ground substance of ECM. GAGs can exist as free chains such as hyaluronan or be covalently linked to core proteins to form proteoglycans, such as CS proteoglycan (CSPG) and heparin sulfate proteoglycan (HSPG)^3,4^. CS is the most abundant GAG in the central nervous system (CNS) matrix. CSPGs participate in neural development, axon pathfinding and guidance, nerve plasticity, and regeneration after CNS injury. Excess CS is one of the major components of glial scars at CNS lesion sites. Moreover, aberrant accumulation of CS is also observed in the ECM of glioma. Using chondroitinase ABC or CSPG inhibitors to eliminate CS deposits in CNS lesions promotes functional recovery after nerve injury^5,6^. In glioma, degradation of CS by chondroitinase ABC also enhances temozolomide availability and efficacy of oncolytic virus therapy in murine glioma models^7,8^.

Many extracellular and membrane-associated proteoglycans are thought to promote glioma formation and progression^9^. For instance, the expression of CSPG4 might be an important prognostic factor in glioblastoma multiforme (GBM), where it is associated with enhanced tumor progression and resistance to radiotherapy^10–12^. In addition, GPC1, CD44, and HSPG2 may promote glioma invasion, development, and angiogenesis^13–16^. Blocking GAGs formation by targeting UDP-Glucose 6-dehydrogenase was recently reported to inhibit GBM growth and migration^17^. Mechanically, both GAGs on proteoglycans and core proteins play critical roles in regulation of cellular signaling and cell migration through their interaction with extracellular ligands, growth factor receptors, ECM components, and intracellular enzymes^3,9^. According to The Cancer Genome Atlas (TCGA) (http://cancergenome.nih.gov/), many CS synthases and sulfotransferases are upregulated in human glioma tissue^9,18^, but comprehensive investigations for the alterations of these GAG elongation and modification enzymes are still scarce. One previous study reported that SULF2, a HS sulfatase, regulates Platelet Derived Growth Factor Receptor Alpha (PDGFRA) signaling and tumor growth in glioma^19^, whereas our recent findings indicate that Dermatan Sulfate Epimerase (DSE), a CS epimerase, is upregulated in glioma, and that it could regulate Erb-B2 Receptor Tyrosine Kinase 2 (ERBB2) signaling in cancer cells^20^. Therefore, aberrant expression of GAG synthases could be involved in glioma progression through dysregulation of critical signaling pathways. A further investigation of the role of biosynthetic enzymes of CS chains in glioma might identify novel molecular targets for its treatment.

The biosynthesis of CS chains begins with the formation of a link between *N*-acetylgalactosamine (GalNAc) and a common tetrasaccharide structure at a serine residue on the core protein. The next step (polymerization) is catalyzed by a group of bifunctional enzymes that have β1–3 glucuronosyltransferase and β1–4 *N*-acetylgalactosaminyltransferase activities. A single CS chain can consist of up to 50 repeating GlcA-GalNAc subunits, which are modified with sulfate groups at various positions. The CS disaccharides are classified according to the modifications they bear^3,4,21^. For example, C5 epimerases (DSE and DSEL) convert certain CS subunits into dermatan sulfate (DS, IdoA-GalNAc) forming CS/DS hybrid chains. Through sulfotransferase catalyzation, CS is mainly *O*-sulfated at C-4 (4-*O*-sulfated) and/or C-6 (6-*O*-sulfated) of GalNAc residue, C-2 of IdoA residue, and occasionally at C-2 of GlcA residue. Depending on the spectrotemporal expression of the polymerization and modification enzymes, a single CS chain usually consists of a series of variably sulfated units.

In this study, we focus on the CS polymerization enzymes that build the elemental structure of CS. We evaluate the correlation between clinicopathological features and expression of CS synthases in glioma patients. We explore the changes of cell surface CS and its contribution to malignant growth of glioma cells, by manipulating CS Synthase 1 (CHSY1) expression. Importantly, we demonstrate that CHSY1 selectively modulates PDGFRA signaling, and that survival of a mouse model of a CHSY1-expressing tumor is increased by using a PDGFR inhibitor.

## Results

### CHSY1 is frequently upregulated in glioblastoma and correlates with high tumor grade and poor survival

**Fig. 1:**
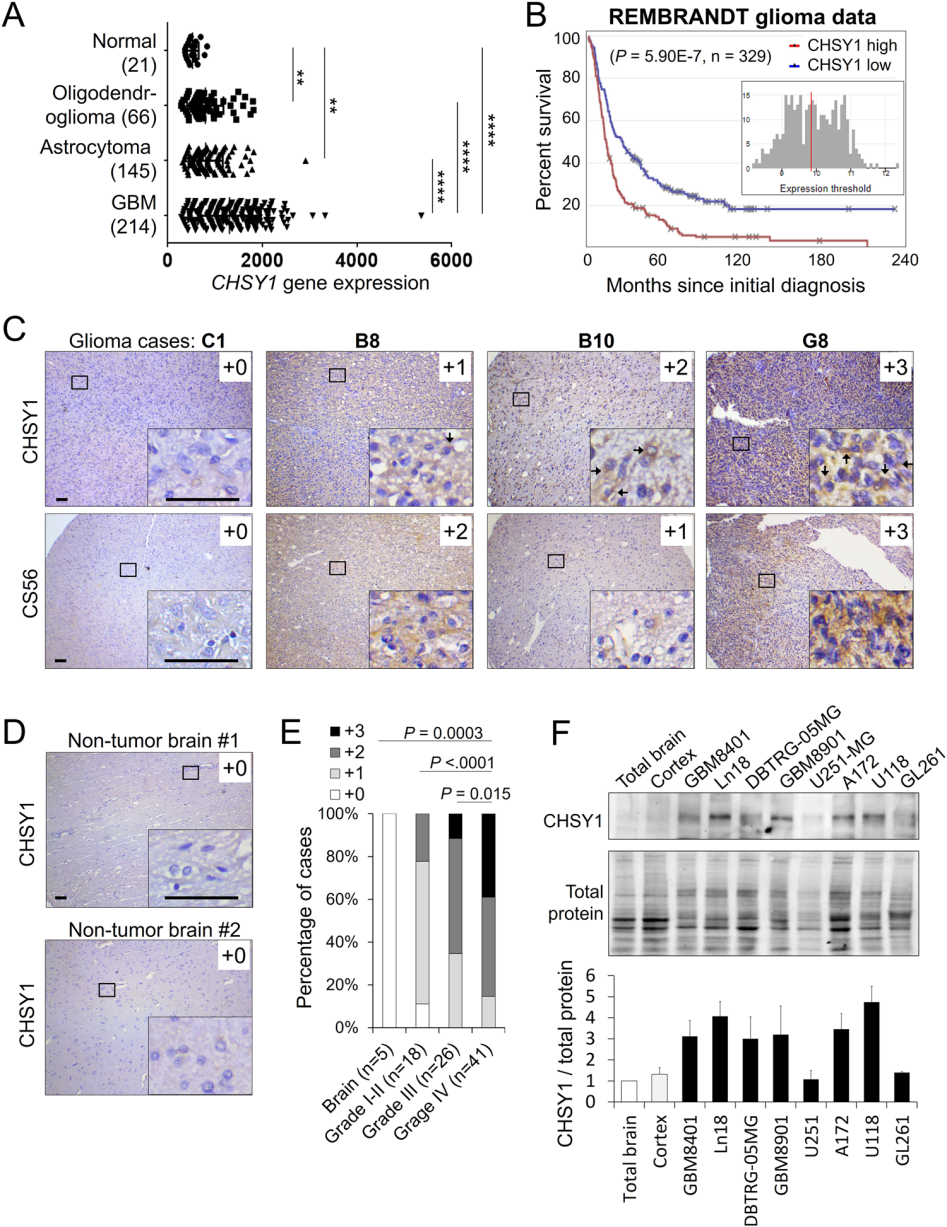
CHSY1 is frequently upregulated in human glioma. **a** Comparison of *CHSY1* gene expression in glioma subtypes and normal brain tissue in the REMBRANDT glioma microarray database. **P < 0.01, ****P < 0.0001. **b** High expression of *CHSY1* was associated with worse overall survival in glioma patients. The high and low expression groups were divided by median expression level of *CHSY1* in 329 cases. These data were from the REMBRANDT database (http://www.betastasis.com/glioma/rembrandt/). **c** Immunohistochemistry of CHSY1 (upper panel) and CS56 (lower panel) on tissue array contains 85 primary glioma cases. The staining was visualized in brown color with a 3,3-diaminobenzidine liquid substrate system. All sections were counterstained with hematoxylin. Representative images of four glioma cases with different staining intensities are shown. Amplified images are shown at the bottom right of each image. Scale bars, 50 μm. Arrows indicate positive stained glioma cells. **d** Representative images of CHSY1 staining on normal brain tissue (*n* = 5). **e** Statistical analysis of immunohistochemistry in glioma tissue array. Mann–Whitney *U*-test was used. *P*-values are shown at top. **f** Expression of CHSY1 in glioma cell lines and normal human brain tissue. The protein expression was analyzed by western blotting. Total loading protein is shown at bottom. Relative expression levels to total brain tissue form three independent blots are shown at the right.

Three bifunctional CS synthases, CHSY1, CHPF (CHSY2), and CHSY3, control polymerization of CS chains. We analyzed REpository for Molecular BRAin Neoplasia DaTa (REMBRANDT) and ONCOMINE databases to compare the expression of these genes in normal brain and in different subtypes of glioma, to elucidate the clinical significance of CS synthases in this disease. Out of the three genes, only CHSY1 was consistently upregulated in GBM compared with normal brain tissue. In addition, its level was significantly higher in GBM than in astrocytoma or oligodendroglioma in all searched datasets (Fig. 1a and Supplementary Fig. S1A, B). According to REMBRANDT database, high expression of CHSY1 is associated with poor overall survival in glioma patients (*n* = 329, *p* = 5.9E – 7) (Fig. 1b). To examine CHSY1 protein expression in human glioma, we carried out immunohistochemistry on glioma tissue array containing 85 primary glioma tissues. CHSY1 was expressed in the paranuclear cytoplasm of the majority of glioma tissues (Fig. 1c). The intensity of staining was scored according to the percentage of CHSY1-positive cells in each sample (0, negative; +1, <20%; +2, 20–50%; +3, >50%). In addition, anti-CS antibody (CS56) was used to reveal corresponding CS intensity in a serial tissue array section. The staining was scored according to the percentage of CS56-positive areas in each sample (0, negative; +1, <20%; +2, 20–50%; +3, >50%; Fig. 1c). Spearman’s rank test revealed that expression of CHSY1 was positively correlated with CS56 intensity (*p* = 1.22E − 12, *r*_s_ = 0.676), indicating that CHSY1 is one of the mediators of CS56 immunoreactivities in glioma tissue. Intriguingly, under our staining conditions, no obvious CHSY1 signal was observed in the normal brain tissue (*n* = 5, Fig. 1d), whereas 85% of grade IV GBM tissues expressed high levels (+2 and +3) of CHSY1, which is significantly higher than that in low-grade glioma and normal brain tissue (Fig. 1e and Supplementary Table S1). When CHSY1 levels in normal human barin tissue and in GBM cell lines were examined, we found that six out of seven human GBM cell lines expressed relatively higher levels of CHSY1 than normal brain tissue, and endogenous mouse Chsy1 in GL261 cells is relatively low (Fig. 1f). Together, these data suggest that CHSY1 is frequently upregulated in glioma patients, and that its expression correlates with the worst histologic grade and poor overall survival.

### CHSY1 mediates CS formation in glioma cells

**Fig. 2:**
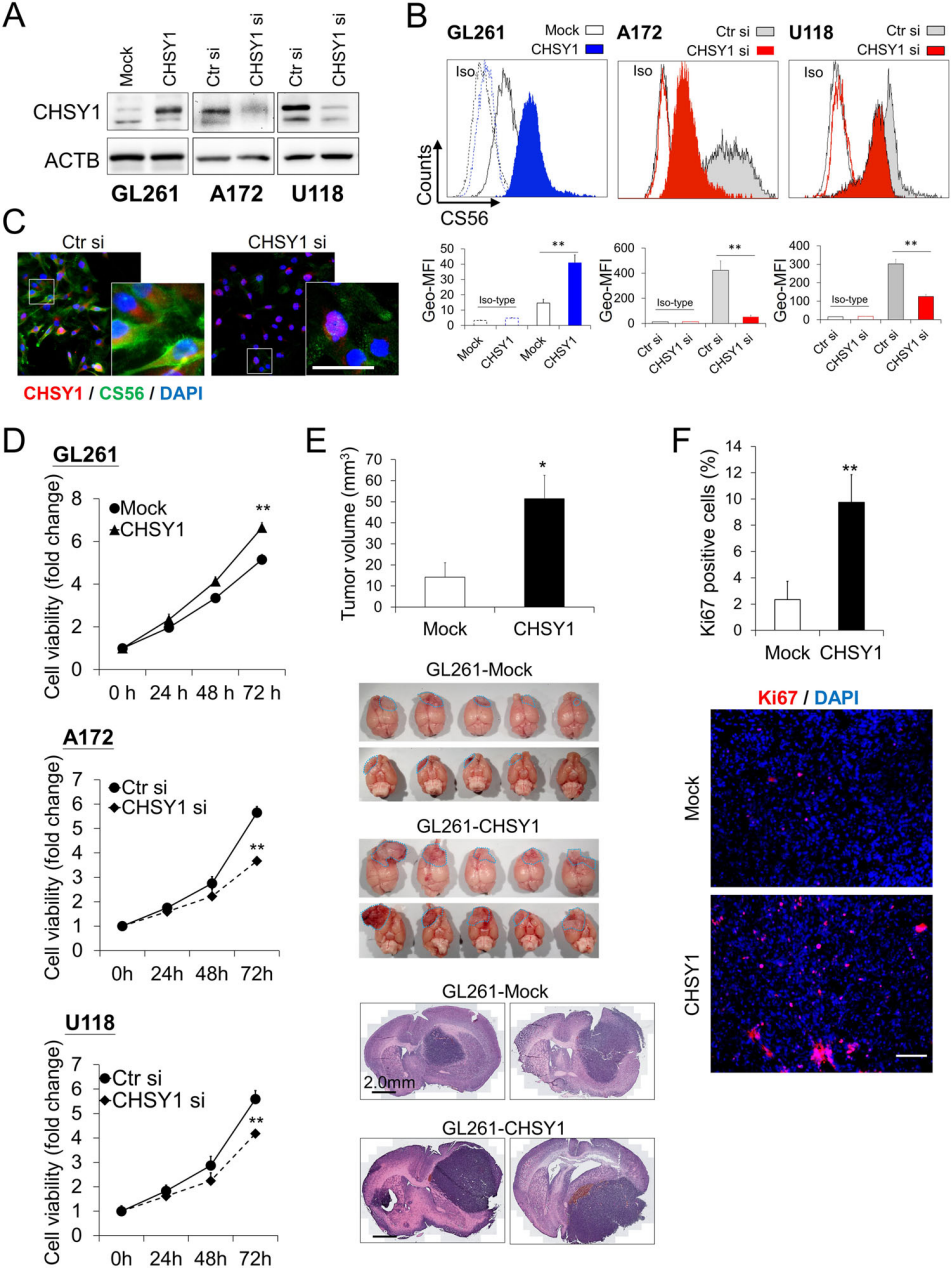
Effects of CHSY1 on chondroitin sulfate formation and malignant growth of glioma cells. **a** Stable overexpression of CHSY1 in GL261 cell and siRNA knockdown of CHSY1 in A172 and U118 cells. The CHSY1 expression were analyzed by western blots. ACTB was used as loading control. Control-siRNA (Ctr si) CHSY1-siRNA (CHSY1 si). **b** Surface CS56 antibody staining on GL261, A172, and U118 transfectants were analyzed by flow cytometry with anti-mouse IgM-FITC. Nonspecific mouse IgM was used as an iso-type control (iso). Representative images are shown. Results of CS56 flow cytometry are presented as the geometric mean fluorescence intensity (Geo-MFI) ± SD from three experiments. **P < 0.01. **c** Immunofluorescence microscopy analysis of CHSY1 expression (red) and CS56 immunoreactivity (green) in control and CHSY1 siRNA-transfected A172 cells. Amplified images are shown at the right. Nuclei were counterstained with DAPI (blue), scale bar, 25 μm. **d** CHSY1 modulated cell viability in vitro. Cell viability of GL261, A172, and U118 cells was measured using CCK8 assays at indicted time points. Data were represented as means ± SD from three independent experiments. **P < 0.01. **e** Overexpression of CHSY1-enhanced tumor growth in vivo. GL261 transfectants were orthotopically injected into right cerebral hemisphere C57BL/6 mice (*n* = 5 for each group). Mouse brain was excised and the size of tumors was measured and shown as means ± SD. *P < 0.05. Gross brain images of superior view and inferior view are shown (middle). Blue dash lines indicate location of tumors. Represented images of H-E stain of brain section are shown. Scale bar, 2.0 mm. **f** CHSY1 promotes cell proliferation in GL261 tumor. Cell proliferation of tumor cells was evaluated by immunofluorescence staining for Ki67 and representative images of tumor tissue of brain section are shown (bottom). Results are presented as the mean ± SD from three fields of each section. **P < 0.01. Scale bar 100 μm.

To investigate the role of CHSY1 in CS formation in glioma cells, CHSY1 overexpression and knockdown experiments with CS56 antibody staining were carried out. As GL261 cells are tumorigenic in C57BL/6 mice and express low levels of CHSY1, we used this cell line for the overexpression experiments. Mock and stable CHSY1 transfectants were obtained from the pooled colonies of GL261 cells transfected with pcDNA3.1 or CHSY1/pcDNA3.1 plasmids, respectively. CHSY1 was silenced by small interfering RNA (siRNA) transfection in A172 and U118 cells, which expressed high levels of CHSY1 (Fig. 2a). The CHSY1-modulated CS formation was quantified by flow cytometry using CS56 staining. We found that CS56 intensity was significantly increased in CHSY1-overexpressing GL261 cells and was decreased in CHSY1-silenced A172 and U118 cells (Fig. 2b). To further examine localization of CHSY1 and CS in glioma cells, immunofluorescence microscopy was used on control and CHSY1-silenced A172 cells. Results showed that CHSY1 was mainly expressed in the paranuclear cytoplasm and strong CS56 staining in the extracellular space, plasma membrane, and cytoplasm of control cells. In contrast, both CHSY1 expression and CS56 staining were dramatically decreased in CHSY1-silenced cells (Fig. 2c). These data indicate that CHSY1 is a crucial enzyme to modulate CS formation in GBM cells in vitro.

### CHSY1 regulates proliferation of glioma cells in vitro and in vivo

Cell Counting Kit-8 (CCK8) was used to measure the effects of CHSY1 overexpression or knockdown on malignant growth of GBM cells. Our data demonstrated that overexpression of CHSY1-enhanced viability of GL261 cells, whereas its knockdown significantly suppressed viability of A172 and U118 cells (Fig. 2d). To analyze the effects of CHSY1 on tumor growth in vivo, mock and CHSY1-overexpressing GL261 cells were transplanted orthotopically into mouse cerebrum. Our results showed that overexpression of CHSY1 significantly increased tumor volume in the 3 weeks after transplantation (Fig. 2e). Immunostaining of excised tumor sections revealed an increased percentage of Ki67-positive cells in CHSY1-overexpressing tumor tissue (Fig. 2f). These data indicate that CHSY1 could promote GBM cell growth by accelerating cell proliferation.

### CHSY1 modulates PDGFRA signaling and stability in glioma cells

**Fig. 3:**
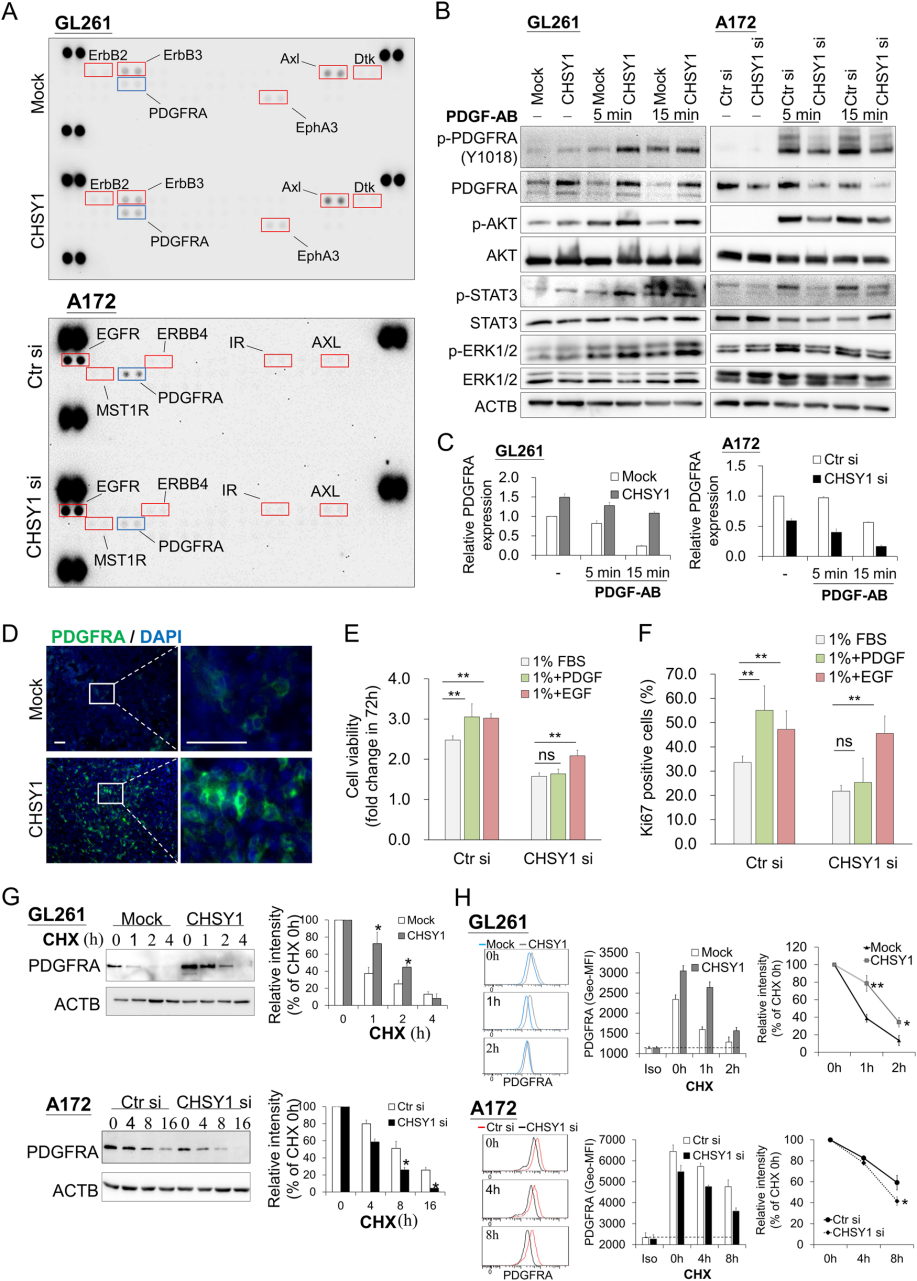
CHSY1 regulates PDGFRA signaling and stability in glioma cells. **a** p-RTK array showing the effects of CHSY1 on the phosphorylation of RTKs. Cells were starved for 3 h and then stimulated with FBS (10%) for 15 min. Cell lysates of mock and CHSY1-overexpressed GL261 cells (left); control and CHSY1-silenced A172 cells (right) were applied to p-RTK arrays including 39 RTKs. **b** CHSY1 modulates PDGF-induced signaling in glioblastoma cells. GL261 and A172 transfectants were starved for 3 h and then treated with (+)/without (−) PDGF-AB (20 ng/mL) for 5 and 15 min. Cell lysates (20 μg) were analyzed by western blotting with various antibodies, as indicated. **c** CHSY1 regulates PDGFRA protein levels in glioblastoma cells. Relative PDGFRA levels of western blottings with or without PDGF-AB stimulation are quantified and shown as mean ± SD from three experiments. **d** Immunofluorescence microscopy analysis of PDGFRA expression (green) in GL261 brain tumor sections. Amplified images are shown at the right. Nuclei were counterstained with DAPI (blue), scale bar, 60 μm. **e** Knockdown of CHSY1 suppresses PDGF-induced cell viability. Control and CHSY1-silenced A172 cells were cultured in low-serum condition (1% of FBS) and treated with PDGF (20 ng/ml) or EGF (20 ng/ml). Cell viability was measured by CCK8 assays at. Data were represented as means ± SD from three independent experiments. **P < 0.01; ns: nonsignificant. **f** Knockdown of CHSY1 suppresses PDGF-induced cell proliferation. Cells were cultured in low-serum condition (1% of FBS) and treated with PDGF (20 ng/ml) or EGF (20 ng/ml) for 48 h. Cells were immunofluorescently stained for Ki67 and Ki67-positive cells were counted under a microscope. Results are presented as means ± SD from three independent experiments. **P < 0.01, ns: nonsignificant. **g** Western blot analysis of PDGFRA protein levels in GL261 and A172 transfectants. Cells were treated with 20 μM cycloheximide (CHX) in culture medium at indicated intervals and analyzed by western blotting. Intensities of PDGFRA protein were quantified using a densitometer. **h** Effects of CHSY1 on surface expression of PDGFRA in GL261 cells and A172 cells. Surface PDGFRA staining after treating cycloheximide at indicated intervals were analyzed by flow cytometry. Nonspecific rabbit IgG was used as an iso-type control (iso). Representative images are shown (left). Statistic results are shown as Geo-MFI ± SD from three experiments (middle). To quantify PDGFRA turnover rate, Geo-MFI of iso-type control was subtracted and the relative PDGFRA intensities to the 0 h of each group are shown at the right. *P < 0.05, **P < 0.01.

In human glioma, aberrant activation of receptor tyrosine kinase (RTK) pathway is the most common alteration^22–24^. CS chains are known to interact with several growth factors and regulate RTK signaling^25^. Thus, we used phospho-RTK antibody arrays to examine whether CHSY1 regulates RTK activities in GBM cells. Interestingly, our data demonstrated that only phospho-PDGFRA was obviously increased in CHSY1-overexpressing GL261 cells. In contrast, knockdown of CHSY1 decreased phosphorylation of PDGFRA (Fig. 3a). Weak and undetectable phospho-RTK in RTK arrays were listed in Supplementary Table S2. To confirm these results, PDGFRA activation and downstream signaling were analyzed by western blottings. Our data showed that overexpression of CHSY1 increased Platelet Derived Growth Factor (PDGF)-induced phosphorylation of PDGFRA, AKT, STAT3, and ERK in GL261 cells, whereas its knockdown decreased PDGFRA phosphorylation and downstream signaling in A172 cells (Fig. 3b). Notably, we found that expression of PDGFRA increased in CHSY1-overexpressing GL261 cells and decreased in CHSY1-silencing A172 cells, before PDGF stimulation. Further, overexpression of CHSY1 inhibited PDGF-triggered decrease in PDGFRA levels, whereas CHSY1 knockdown accelerated PDGFRA decrease following PDGF stimulation (Fig. 3c). Moreover, immunofluorescence microscopy revealed that PDGFRA was highly expressed on the cell membrane of CHSY1-overexpressing GL261 tumor tissue sections (Fig. 3d). To measure the effects of CHSY1 on growth factor-induced cell growth and proliferation, control and CHSY1-silienced A172 cells were treated with PDGF and Epidermal Growth Factor (EGF) in low-serum culture condition (1% fetal bovine serum (FBS)); both PDGF and EGF promoted cell viability and increased Ki67-positive cells in control A172 cells, whereas knockdown of CHSY1 curtailed the PDGF-mediated increase in cell viability and proliferation (Fig. 3e, f and Supplementary Fig. S2).

We used quantitative PCR to elucidate the mechanism that leads to changes in PDGFRA protein levels. We found that altering CHSY1 expression had no influence on the level of PDGFRA mRNA in GL261 and A172 cells (Supplementary Fig. S3A). In agreement with these findings, search of REMBRANDT and TCGA databases found no association between gene level of CHSY1 and PDGFRA expression in human glioma tissue (Supplementary Fig. S3B). We next sought to determine whether CHSY1 affects PDGFRA stability. In the presence of cycloheximide, a protein synthesis inhibitor, PDGFRA turnover was slower in CHSY1-overexpressing cells than in mock cells. In contrast, PDGFRA turnover in CHSY1-silenced cells was faster than in control cells (Fig. 3g). Similar results were obtained for cell surface PDGFRA by using flow cytometry (Fig. 3h). Together, these results suggest that CHSY1 selectively regulates the PDGFRA pathway and enhances PDGFRA protein stability in GBM cells.

### PDGFRA inhibition reverses CHSY1-mediated tumor growth in vitro and in vivo

**Fig. 4:**
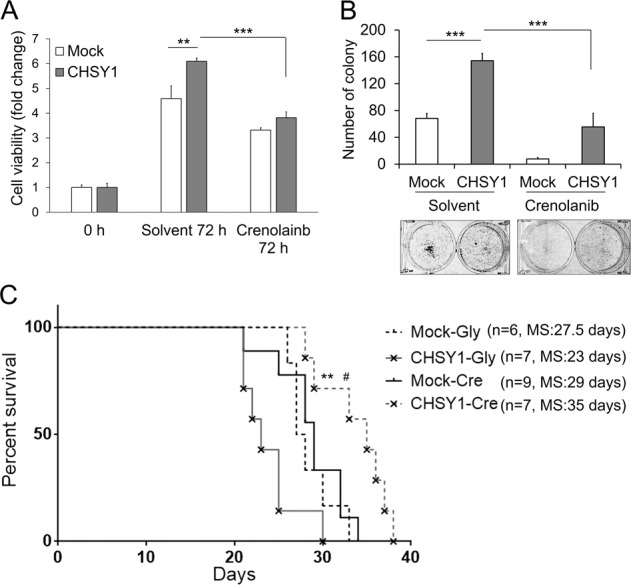
PDGFRA antagonist suppresses CHSY1-mediated malignant growth. **a** Effects of crenolanib, a PDGFRA inhibitor, on CHSY1-enhanced cell viability. GL261 transfectants were treated with solvent (0.005% of ethanol) or crenolanib (0.5 μM) and then analyzed by CCK8 assays at 72 h. Data are represented as means ± SD from three independent experiments. **P < 0.01, ***P < 0.001. **b** Effects of PDGFRA inhibitor on anchorage-dependent colony formation. Results were presented as means ± SD from three independent experiments. Representative images were shown at the bottom. ***P < 0.001. **c** Survival analysis of crenolanib treatment in an orthotopic murine model of GL261 glioma. Mock and CHSY1-overexpressing transfecants were implanted into C57BL/6 mice. The next day post tumor implant, mice were treated with 15 mg/kg of crenolanib (Cre) via daily intraperitoneal injection for 7 days. Equivalent 5% of glycerol formal (Gly) was taken as solvent control. Survival was assessed via Kaplan–Meir survival curves. **P < 0.01 (CHSY1-Gly vs. CHSY1-Cre*)*. ^#^*P* < 0.05 (Mock-Cre vs. CHSY1-Cre). Median survival time (MS) of each group is shown at the right.

We used CCK8 and colony formation assay to assess whether crenolanib, a PDGFRA inhibitor, could reverse CHSY1-mediated malignant growth of GBM cells. The results showed that crenolanib (Cre) significantly decreased cell viability and colony formation in CHSY1-overexpressing GL261 cells (Fig. 4a, b).

To evaluate the therapeutic effect of PDGFRA inhibition on tumor growth in vivo, we administered Cre (15 mg/kg) or 5% glycerol formal (Gly, solvent) to an orthotopic murine model of GL261 glioma for 7 days. The mice experienced no significant body weight loss during these treatments (data not shown). The median survival time was 27.5, 29, 23, and 35 days in the mock-Gly group (*n* = 6), mock-Cre group (*n* = 9), CHSY1-Gly group (*n* = 7), and CHSY1-Cre group (*n* = 7), respectively. Cre treatment significantly extended the median survival time in the CHSY1-overexpressing group (CHSY1-Cre vs. CHSY1-Gly, *p* = 0.0018; CHSY1-Cre vs. mock-Cre, *p* = 0.0117), whereas no survival benefit was observed in the mock-Gly and mock-Cre group (Fig. 4c). These data suggest that blocking PDGFRA activities can inhibit CHSY1-induced malignant growth of GBM cells.

## Discussion

Glioblastoma is one of the most aggressive human cancers and the current median survival time of GBM patients is less than 2 years. The high mortality of GBM is mainly attributable to its inevitable recurrence after surgical resection of the primary tumor and limited knowledge of targetable genetic aberrations^26,27^. To develop novel strategies for treating this fatal disease, we explored the role of CHSY1 in glioma and its potential as biomarker. CHSY1 expression in glioma tumors is positively associated with adverse clinicopathologic factors and is a predictor of poor survival. CHSY1 promotes malignant growth in multiple GBM cell lines in vitro and in a mouse orthotopic glioma model. CHSY1-mediated CS formation and PDGFRA activation are thought to underlie this effect. We also propose that CHSY1 stabilizes PDGFRA protein levels. Targeting PDGFR pathway with small molecule inhibitors significantly inhibited CHSY1-mediated GBM tumor growth in murine models.

The remarkable structural diversity of CS chains generates biological information that can be unique to each CS chain and changes in CS structure dictate their binding ability to other molecules. In the CNS, CS composition changes at different stages of development. For example, during embryogenesis, 6-*O*-sulfated CS is the most highly expressed, whereas in adulthood 4-*O*-sulfated CS becomes the most abundant type of CS in the nerve tissue^28,29^. Interestingly, in a vast majority of cancers, a substantial increase in the level of 6‐*O*‐sulfated CS is observed. In some cancers (e.g., renal and gastric cancer), this change is concomitant with a reduction in the 4‐*O*‐sulfated CS^30–32^. CS sulfotransferases, CHST7, CHST3, and CHST11 are upregulated in human glioma, whereas changes to CS sulfation subunits are not well documented^9^. Here we demonstrated that CS56 staining is moderately associated with CHSY1 expression in human glioma tissue (*r*_s_ = 0.676), suggesting that other CS modification enzymes, such as DSE and CHSTs, may also modulate CS56 epitopes. As a previous report suggested that the CS56 monoclonal antibody preferentially binds to 6-*O*-sulfated CS^33^ and a recent gene knock-in experiments revealed that CHST3-mediated 6-*O*-sulfation exclusively induced strong CS56 binding^34^, accordingly, our CS56 staining data partially reflect the quantity of CS and suggests that CHSY1 may mediate the increases of 6-*O*-sulfated CS in human glioma.

Recently, a specific CS-binding protein (VAR2SCA) derived from the malaria parasite was used to identify oncofetal CS expression in many cancer types, which can be taken as a diagnostic marker, circulating tumor cell indicator, or drug target^35,36^. However, diverse composition of CS elongation and modification enzymes in different cell types hinders the identification of crucial modulators involved in oncofetal CS formation. Owing to the GAGOme library developed with CRISPR/Cas9 knockout and knock-in technology, the key enzymes participating in VAR2SCA binding have been identified^34^. The knockout experiments revealed that CHSY1 is an essential CS elongation enzyme required for VAR2SCA binding. Our data indicates that CHSY1 is frequently upregulated in human glioma, particularly in GBM, suggesting the potential for VAR2SCA targeting. CHSY1 is also upregulated in other types of solid tumor, including hepatocellular carcinoma, colorectal cancer, and soft tissue sarcomas^37–39^. These results imply that overexpression of CHSY1 in tumor cells results in CS accumulation in the ECM and may facilitate the formation of oncofetal CS in the tumor microenvironment.

Previous reports show that over 60% of human GBM have at least one amplification and/or mutation in an RTK gene. Alteration of EGFR is the most common, whereas that of PDGFRA is the second most common. Activation of the PDGFR pathway is a key regulator of glioma development and progression^24,40,41^. This study was the first to demonstrate that CHSY1 selectively regulates the PDGFR pathway by modulating PDGFRA protein levels. PDGF stimulation assays and cycloheximide assays revealed that CHSY1 regulates PDGFRA protein stability in GBM cells (Fig. 3), which may result from suppressing PDGFRA endocytosis and/or escaping protein degradation. Accordingly, we propose that CHSY1 upregulation in GBM cells may promote oncogenic addiction to PDGFR signaling. Crenolanib, a PDGFR inhibitor in Phase II trials for glioblastoma with PDGFRA gene amplification, inhibits glioma cells^42^. In our orthotopic glioma model, Cre treatment significantly enhanced survival of mice transplanted with CHSY1-overexpressing cells, whereas no obvious response to the treatment was seen in the mock-transfected group. Interestingly, interaction of CSPG4 with PDGFRA has been shown to promote oligodendrocyte progenitor cells proliferation in response to PDGF^43^ and a HS modification enzyme, SULF2, is also able to mediate PDGFRA activity in glioma cells^19^. Moreover, PDGFs might interact with CS and HS to regulate receptor activation^44–46^. Thus, we believe that CS, whose polymerization is mediated by CHSY1, might act as a co-activator or co-receptor to regulate PDGFRA. Although detailed mechanisms underlying these effects are yet to be elucidated, these findings imply that aberrant expression of GAG synthases, such as CHSY1, could provide a molecular basis for targeting PDGFRA pathway in glioma.

Identification of the specific pathways driving individual tumors is key to improving their treatment. In this study, we showed that CHSY1 is not only a prognostic factor for poor survival of glioma patients but that it also activates the PDGFRA pathway to promote tumorigenesis. In addition to its potential as a therapeutic target in GBM, CHSY1 may help to identify tumors that rely on PDGF signaling.

## Materials and methods

### Cell culture and tranfection

The source and the species of glioma cell lines, GBM8401, GBM8901, DBTRG-05MG, A172, LN18, U118, U251-MG, and GL261, were listed in Supplementary Table S3. For overexpression experiments, empty pcDNA3.1 and CHSY1-pcDNA3.1 plasmids were transfected to GL261 cells using Lipofectamine 2000 (Invitrogen). The transfected cells were selected with 600 μg/mL of G418. For knockdown of CHSY1, ON-TARGETplus SMARTpool siRNA against *CHSY1* and control siRNA were purchased from Dharmacon. Cells were transfected with 20 nmol of siRNA using Lipofectamine RNAiMAX (Invitrogen) for 48–72 h.

### Reagents and antibodies

Full-length CHSY1 cDNA clone and antibody against CHSY1 were purchased from OriGene. CCK8 reagent and cycloheximide were purchased from Sigma-Aldrich. Antibody against Ki67 was purchased from Abcam. Antibodies against p-AKT, AKT, p-STAT3, STAT3, p-ERK1/2, ERK1/2, p-PDGFRA (Y1018), and PDGFRA were purchased from Cell Signaling Technology. Antibodies against CS (CS56) and ACTB were purchased from GeneTex, Inc. Recombinant PDGF-AB and EGF were purchased from PeproTech. Cre was purchased from Cayman Chemical.

### Tissue array and immunohistochemistry

Paraffin-embedded human glioma tissue microarrays were purchased from Shanghai Outdo Biotech and Pantomics, Inc. Arrays were incubated with CHSY1 antibody (1:200) in 5% bovine serum albumin/phosphate-buffered saline and 0.1% Triton X-100 (Sigma) for 16 h at 4 °C. UltraVision Quanto Detection System (Thermo Fisher Scientific, Inc.) was used to amplify primary antibody signal. For immunohistochemistry of CS56, biotinylated goat anti-mouse IgM antibody and avidin–biotin complex kit (Vector Laboratories) were used. The specific immunostaining was visualized with 3,3-diaminobenzidine and counterstained with hematoxylin (Sigma). The distribution and positive intensity were graded by microscopy, by two scorers blinded to the clinical parameters. Images were obtained by TissueFAX Plus Cytometer.

### Western blotting and phospho-RTK array assay

Adult normal human brain tissue lysates were purchased from Novus Biologicals. Total protein was measured by stain-free technology (Bio-Rad). To analyze PDGF-triggered signaling, cells were serum starved for 3 h and then stimulated with 20 ng/ml of PDGF-AB for 5 min and 15 min. Intensity of signals on western blottings was quantified by ImageJ software (Wayne Rasband). For phospho-RTK array assay, cells were serum starved for 3 h and then stimulated with FBS (10% in final) for 15 min. Three hundred micrograms of protein lysate were applied to phospho-RTK array Kit (R&D Systems^TM^) according to the manufacturer’s protocol.

### Flow cytometry for cell surface antigen expression

GBM cells were detached with 10 mM EDTA and stained with CS56 antibody at 1∶100 dilutions on ice for 30 min. Cells were incubated with fluorescein isothiocyanate (FITC)-conjugated anti-mouse IgM antibody on ice for 30 min. For measuring cell surface PDGFRA expression, cycloheximide-treated cells were detached and immediately fixed with 4% paraformaldehyde for 15 min. Cells were stained with PDGFRA antibody at 1∶200 dilutions. A FITC-conjugated anti-rabbit IgG was used as the secondary antibody. Florescence intensity was analyzed by FACScan cytometer (BD Pharmingen).

### Cell viability and colony formation

Cells (2 × 10^3^) were seeded into 96-well plates with culture medium. Cell viability was analyzed by CCK8 assay at 0, 24, 48, and 72 h following the manufacturer’s protocol (Sigma-Aldrich). In brief, four wells per group of each time point were measured by OD 450 nm and two wells of only media were used to measure the background absorbance. The experiments were repeated for three times and relative fold changes were shown. For anchorage-dependent colony formation assay, 500 cells were seeded in 6-well plates. Colonies were stained by crystal violet and counted after 14 days incubation.

### Animal experiments

Orthotopic glioma model was used for the evaluation of CHSY1-mediated malignant growth and treatment effects of PDGFRA inhibitor, crenolanib. Eight-week-old male C57BL/6 mice were purchased from National Laboratory Animal Center (Tainan, Taiwan). GL261 mock transfectants (1 × 10^5^) and CHSY1 transfectants in 2 µl of Dulbecco’s modified Eagle’s medium were injected into the right cerebral of mice using a stereotactic device (coordinate: 1 mm posterior and 2 mm lateral from bregma, and 2.5 mm depth from the dura). Tumor growth was measured after 3 weeks. Mice were killed and perfused with 4% paraformaldehyde under anesthesia. The brains were dissected for size examination and immunostaining. The largest (*a*) and the smallest (*b*) diameters of the tumors were measured, and tumor volume was calculated using the formula: *a* × *b*^2^ × 0.4. For survival analysis, mice were intraperitoneally injected with 15 mg/kg of Cre or equivalent solvent and 5% of Gly (Sigma-Aldrich), on the day after GL261 transplantation for 7 serial days. The usage of Cre in our animal model was by referring to previous reports^47,48^. Mice were observed daily and were killed when they reached endpoints, including loss exceeding 20% of body weight or hunched posture. All animal experiments in this study were reviewed and approved by the Institutional Animal Care and Use Committee of Chung Shan Medical University Experimental Animal Center.

### Statistical analysis

Data analysis was performed using GraphPad Prism 6. One-way analysis of variance multiple comparisons were used for analyzing *CHSY1* gene expression in different glioma subtypes. Student’s *t*-test was used for statistical analyses. Spearman’s rank correlation was used to analyze CHSY1 and CS56 staining intensities (https://www.wessa.net/). Mann–Whitney *U*-test was used to compare intensity of immunohistochemistry on normal brain and glioma tissues. Two-sided Fisher’s exact test was used for comparisons between CHSY1 expression and clinicopathologic features of glioma tissue array. Kaplan–Meier analysis and the log-rank test were used to estimate survival rate. *P* < 0.05 was considered statistically significant.

## Supplementary information

Table S1

Table S2

Table S3

Figure S1

Figure S2

Figure S3
