# Supplementary material for: Chondroitin sulfate synthase 1 enhances proliferation of glioblastoma by modulating PDGFRA stability
Source: Oncogenesis. 2020 Feb 4;9(2):9. doi: 10.1038/s41389-020-0197-0 (PMC7000683; doi:10.1038/s41389-020-0197-0)
Supplement: Supplementary file 1 — Table S1 [file 41389_2020_197_MOESM1_ESM.docx]

**Table S1. Correlation of CHSY1 expression with clinicopathological features of glioma tissue array.**

|  |  | **CHSY1 expression** | |  |
| --- | --- | --- | --- | --- |
| **Factor** |  | **Low**  **(0 and +1)** | **High**  **(+2 and +3)** | ***P* value (Two-sided Fisher's exact test)** |
| Tissue types | Non-tumor | 5 | 0 | 0.0063* |
|  | Tumor | 29 | 56 |  |
| Sex^#^ | Male | 8 | 32 | 0.0079* |
|  | Female | 18 | 18 |  |
| Age^#^ | < 55 years | 13 | 17 | 0.2192 |
|  | ≥ 55 years | 13 | 33 |  |
| Tumor stage | Grade I – III^$^ | 23 | 21 | 0.0003* |
|  | Grade IV (GBM) | 6 | 35 |  |

**P* < 0.05 was considered as statistically significant.

^#^Night patients’ sex and age were not provided.

^$^Astrocytoma and Oligodendroglioma.
