# Supplementary material for: Chondroitin sulfate synthase 1 enhances proliferation of glioblastoma by modulating PDGFRA stability
Source: Oncogenesis. 2020 Feb 4;9(2):9. doi: 10.1038/s41389-020-0197-0 (PMC7000683; doi:10.1038/s41389-020-0197-0)
Supplement: Supplementary file 2 — Table S2 [file 41389_2020_197_MOESM2_ESM.docx]

**Table S2. Weak and undetectable phospho-RTK in RTK array**

| **Cell line** | **Weak and undetectable phospho-RTK in RTK array in FBS treated condition** |
| --- | --- |
| GL261 | EGFR, ErbB4, Fgfr2, Fgfr3, Fgfr4, IR, IGF-1R, Mer, Met, Mst1R, PDGFRB, SCFR, Flt3, M-CSFR, c-Ret, Tie-1, Tie-2, TrkA, TrkB, TrkC, VEGFR1, VEGFR2, VEGFR3, Musk, EphA1, EphA2, EphA6, EphA7, EphA8, EphB1, EphB2, EphB4, EphB6, |
| A172 | ERBB2, ERBB3, FGFR2, FGFR3, FGFR4, DTK, IGF-1R, MER, MET, PDGFRB, SCFR, FLT3, M-CSFR, c-RET, TIE-1, TIE-2, TRKA, TRKB, TRKC, VEGFR1, VEGFR2, VEGFR3, MUSK, EPHA1, EPHA2, EPHA3, EPHA6, EPHA7, EPHA8, EPHB1, EPHB2, EPHB4, EPHB6, |
