# Supplementary material for: Chondroitin sulfate synthase 1 enhances proliferation of glioblastoma by modulating PDGFRA stability
Source: Oncogenesis. 2020 Feb 4;9(2):9. doi: 10.1038/s41389-020-0197-0 (PMC7000683; doi:10.1038/s41389-020-0197-0)
Supplement: Supplementary file 3 — Table S3 [file 41389_2020_197_MOESM3_ESM.docx]

**Table S3.** Information of glioma cell lines.

| **Name** | **Collection Number.** | **Domain** | **Source** |
| --- | --- | --- | --- |
| Ln18 | ATCC, CRL-2610 | Cell Line | Homo sapiens (human) brain; glioblastoma; glial cell human glioblastoma |
| DBTRG-05MG | Bioresource Collection and Research Center (BCRC), Taiwan, BCRC Number: 60380 | Cell Line | Homo sapiens (human) brain; glioblastoma; glial cell human glioblastoma |
| U251-MG | Kerafast, Inc. Number: EDK001 | Cell Line | Homo sapiens (human) brain; glioblastoma; mutilforme Human brain glioblastoma multiforme |
| A172 | ATCC, CRL-1620 | Cell Line | Homo sapiens (human) brain; glioblastoma |
| U118 (U-118 MG) | ATCC, HTB-15 | Cell Line | Homo sapiens (human) brain; glioblastoma |
| GBM8401 | Obtained from Dr. Wei-Hwa Lee, Tri-service General Hospital, Taipei, Taiwan.  BCRC Number: 60163 | Cell Line | Homo sapiens (human) brain; glioblastoma; multiforme Human brain glioblastoma multiforme |
| GBM8901 | Obtained from Dr. Wei-Hwa Lee, Tri-service General Hospital, Taipei, Taiwan.  BCRC Number: 60164 | Cell Line | Homo sapiens (human) brain; glioblastoma; mutilforme Human brain glioblastoma multiforme |
| GL261 | Leibniz Institute  DSMZ, Number: ACC 802 | Cell Line | Mus musculus (Mouse) Breed/subspecies: C57BL/6. Mouse glioblastoma |
