# Supplementary material for: Chondroitin sulfate synthase 1 enhances proliferation of glioblastoma by modulating PDGFRA stability
Source: Oncogenesis. 2020 Feb 4;9(2):9. doi: 10.1038/s41389-020-0197-0 (PMC7000683; doi:10.1038/s41389-020-0197-0)
Supplement: Supplementary file 4 — Figure S1 [file 41389_2020_197_MOESM4_ESM.pdf]

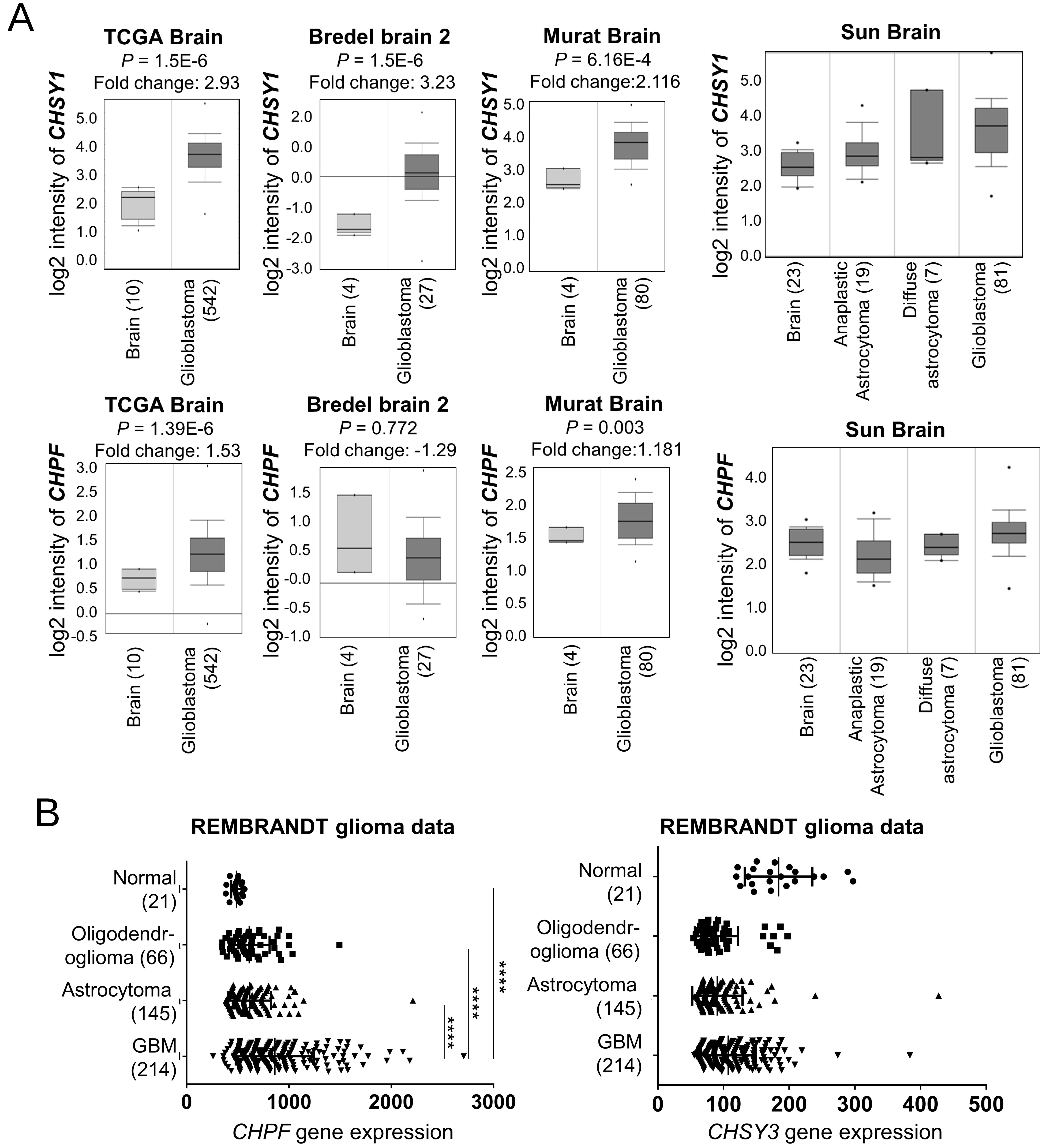

Figure S1. Gene expression of chondroitin sulfate synthase in human glioma. (A) Expression of *CHSY1* and *CHPF* in the ONCOMINE cancer microarray database. Note that Four independent datasets showed that gene expression of *CHSY1* is significantly up-regulated in glioma tissue, compared to normal brain tissue.(B) Comparison of *CHPF* and *CHSY3* gene levels in glioma subtypes and normal brain tissue in the REMBRANDT glioma microarray database. ****P<0.0001.
