# Supplementary material for: Chondroitin sulfate synthase 1 enhances proliferation of glioblastoma by modulating PDGFRA stability
Source: Oncogenesis. 2020 Feb 4;9(2):9. doi: 10.1038/s41389-020-0197-0 (PMC7000683; doi:10.1038/s41389-020-0197-0)
Supplement: Supplementary file 5 — Figure S2 [file 41389_2020_197_MOESM5_ESM.pdf]

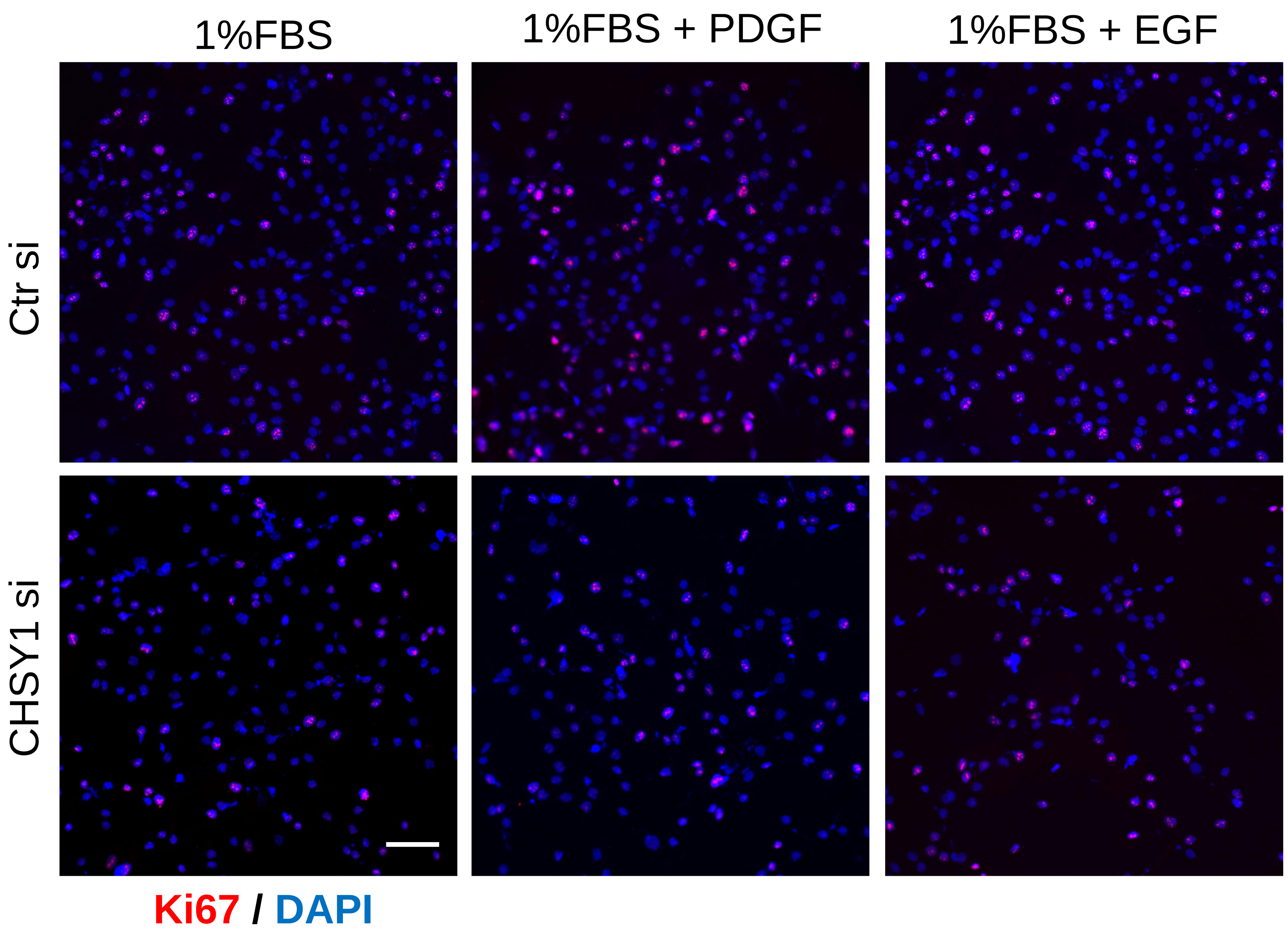

Figure S2. Knockdown of CHSY1 suppresses PDGF-induced cell proliferation. Control and CHSY1 silenced A172 cells were cultured in low serum condition (1% of FBS) and treated with PDGF (20 ng/ml) or EGF (20 ng/ml) for 48 hours. Cells were immunofluorescently stained for Ki67 and Ki67–positive cells were counted under a microscope. Representative images are shown. Scale bar 100 µm
