# Supplementary material for: Chondroitin sulfate synthase 1 enhances proliferation of glioblastoma by modulating PDGFRA stability
Source: Oncogenesis. 2020 Feb 4;9(2):9. doi: 10.1038/s41389-020-0197-0 (PMC7000683; doi:10.1038/s41389-020-0197-0)
Supplement: Supplementary file 6 — Figure S3 [file 41389_2020_197_MOESM6_ESM.pdf]

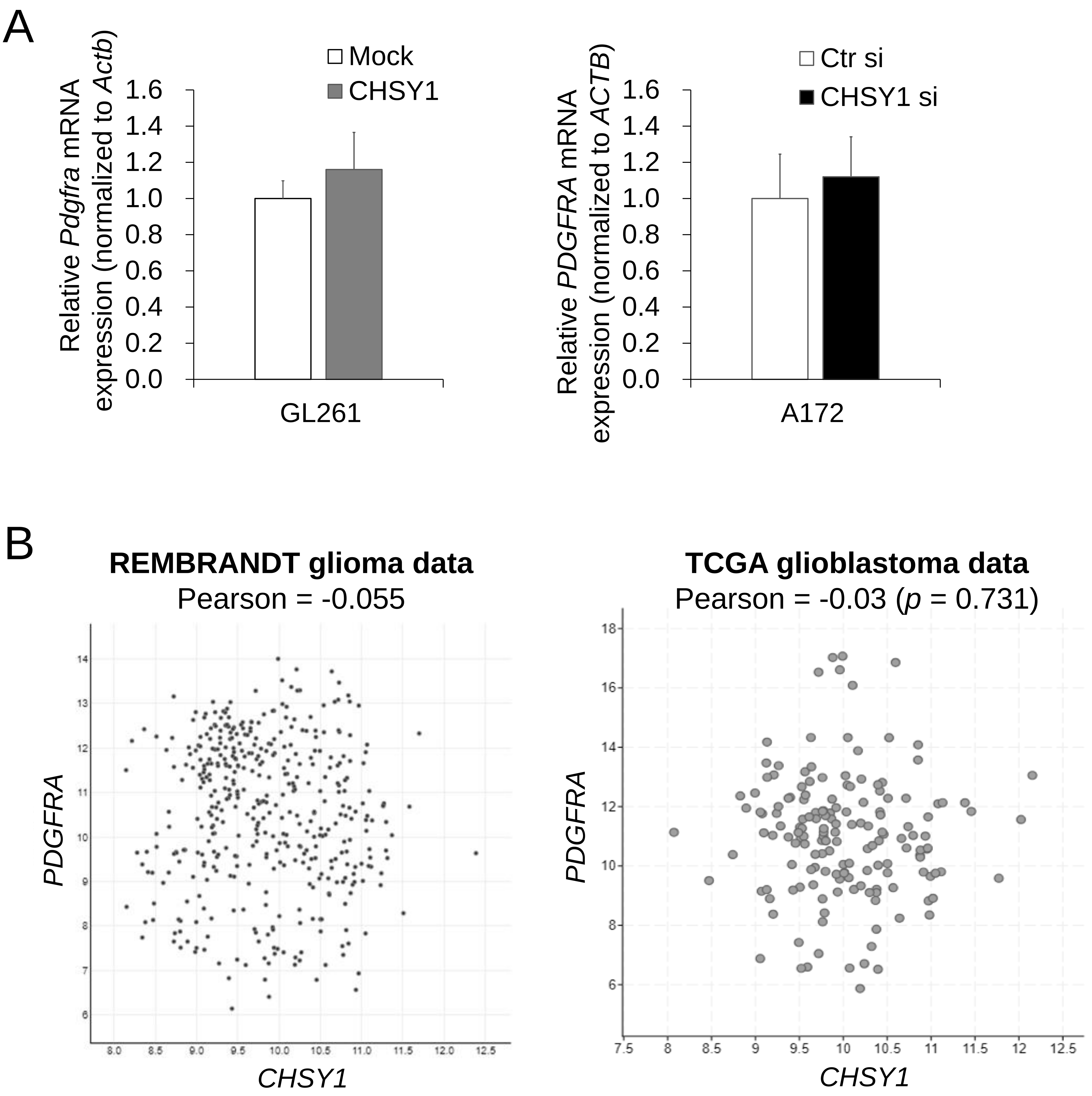

Figure S3. Gene expression of PDGFRA in glioma cells and tumor tissue. (A) Expression of PDGFRA mRNA in mock and CHSY1 overexpressed GL261 and (left), and control and CHSY1 siRNA transfected A172 cells (right). The relative mRNA levels of PDGFRA were analyzed by real-time RT-PCR and normalized to ACTB. (B) Gene expression correlation between *PDFGRA* and *CHSY1* in REMBRANDT glioma dataset (n=425) and TCGA glioblastoma dataset (n=136)
